# Supplementary material for: Selecting High-Performance Gold Nanorods for Photothermal Conversion
Source: Nanomaterials (Basel). 2022 Nov 25;12(23):4188. doi: 10.3390/nano12234188 (PMC9737450; doi:10.3390/nano12234188)
Supplement: Supplementary file 1 [file nanomaterials-12-04188-s001.zip › nanomaterials-2020347-supplementary.pdf]

Selecting High Performance Gold Nanorods for Photothermal Conversion: supplemental document

This supporting document includes experimental data obtained during experimentation and further elaborates on the development of equations presented in the main manuscript.

1. COMPARISON OF ABSORPTION CROSS-SECTIONS OBTAINED BY FEM SIMULATION AND MIE-GANS THEORY

The absorption cross-section obtained by FEM simulations in COMSOL were compared to Mie-Gans theory calculations. To better describe the optical response of larger AuNRs, the Modified Long Wavelength Approximation (MLWA)[1] was employed. This improvement of Mie-Gans theory also includes the effect of radiation damping due to large NR sizes, which is very useful for our applications since we're dealing with a broad range of NR diameters and lengths. Figure S1 shows the comparison among Mie-Gans theory, FEM simulation and the experimental extinction of a 41x10nm AuNR in water. The differences between FEM and Mie-Gans theory may be explained by the actual shape of the nanostructure, since the simulated AuNRs are cylinders capped with hemispheres, while Mie-Gans theory describes spheroids. Nevertheless, this comparison is useful to validate the FEM model, and allowed us proceed with computational simulations. The broadening in experimental extinction occurs due to the presence of a distribution of AuNR sizes in the sample.

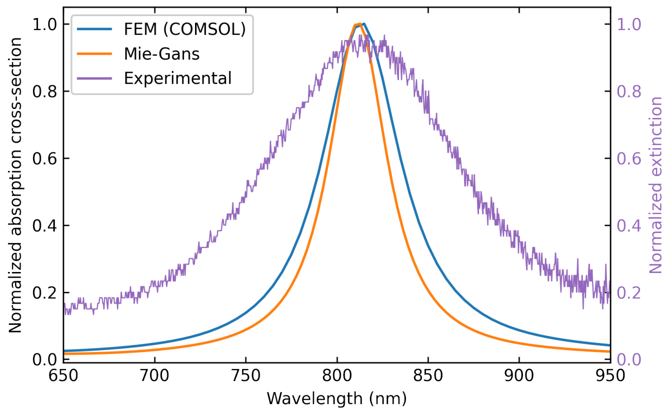

Figure S1. Comparison of the absorption cross-sections obtained by FEM simulations and Mie-Gans theory for 41x10nm AuNR.

2. GOLD NANOROD SAMPLE PREPARATION

Gold nanoparticle samples were acquired from Nanopartz, in which three distinct samples of gold nanorod particles in DI water were acquired. Figure S2 illustrates the optical extinction of each of the acquired sample in UV-Vis spectroscopy in 1cm cuvette.

The samples were diluted in order to have the same number of NPs per volume. The samples characteristics and its dilution for experimental measurements are displayed in Table S1.

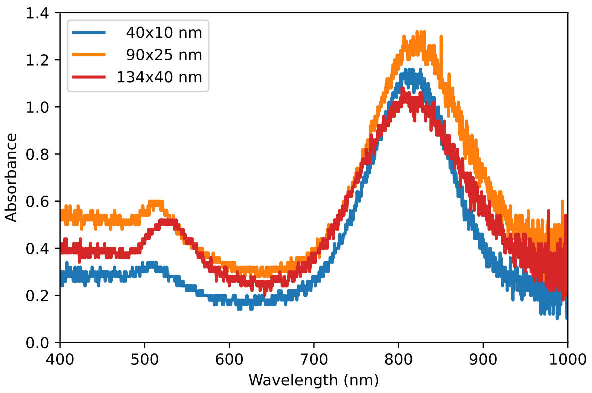

Figure S2. UV-Vis absorbance of the acquired gold nanorod samples. Blue: 40x10nm, Orange: 90x25nm and Red: 134x40nm.

Table S1. Preparation of colloidal NP samples.

AuNR size (nm $\times$ nm)	NP concentration (m^{-3})	Colloid vol. (μL)	DI Water vol. (μL)
40×10	2.3×10^{16}	22	478
90×25	2.5×10^{15}	197	303
134×40	1.0×10^{15}	500	-

3. NANOPARTICLE HEATING BY SINGLE FEMTOSECOND PULSE

To evaluate the temperature dynamics of a single NP under a single fs pulse, we start from the heat equation:

$$\rho_{Au} c_{pAu} \frac{\partial}{\partial t} \left[\Delta T^{fs}_{global}(t) \right] = \kappa_{Au} \nabla^2 \left[\Delta T^{fs}_{global}(t) \right] + \dot{Q}_{in}(t) - \dot{Q}_{out}(t) \,. \tag{S1}$$

The energy balance of the system given by the difference between the power source density and the power loss density, given by $\dot{Q}_{in}(t)$ and $\dot{Q}_{out}(t)$, respectively. Considering a uniform temperature distribution inside the NP, the Laplacian of the temperature change is null. The rate of change of temperature over time ($\Delta T^{fs}_{np}(t)$) is driven by laser absorption and heat conduction to the surrounding medium over a timescale τ_d. The power source density is the heat power transferred from the electron gas to the atomic lattice of the nanoparticle, which is highly dependent of laser pulse duration. If laser pulse is shorter than electron-phonon relaxation, the power source density is given by[2]:

$$\dot{Q}_{in}(t) = \frac{\sigma_{abs} \langle I \rangle}{f \tau_{ep} V_{np}} e^{-t/\tau_{ep}} \,. \tag{S2}$$

Following the Fourier Law, the power loss density is driven by conductive contributions in the NP outward direction that is proportional to the temperature difference between NP and surrounding medium temperature:

$$\dot{Q}_{out}(t) = \rho_{Au} c_{pAu} \frac{\Delta T^{fs}_{np}(t)}{\tau_d} \,. \tag{S3}$$

Therefore, The heat equation becomes:

$$\frac{\mathrm{d}}{\mathrm{d}t} \left[\Delta T^{fs}_{np}(t) \right] + \frac{1}{\tau_d} \Delta T^{fs}_{np}(t) = \frac{\langle I \rangle}{\rho_{Au} c_{pAu} f} \frac{\sigma_{abs}}{V_{np}} \frac{e^{-t/\tau_{ep}}}{\tau_{ep}} . \tag{S4}$$

Exploring the integrating factor method to solve the ODE described by Equation S4, we get a simple analytical solution for the temperature of a single NP:

$$\Delta T_{np}^{fs}(t) = \frac{\langle I \rangle}{\rho_{Au} c_{pAu} f \left(1 - \tau_{ep}/\tau_d\right)} \frac{\sigma_{abs}}{V_{np}} \left[e^{-t/\tau_d} - e^{-t/\tau_{ep}} \right]. \tag{S5}$$

Figure S3 shows the temperature dynamics of different single gold nanorods of different sizes. Notice how bigger NP volumes leads to longer relaxation times and lower temperature build up.

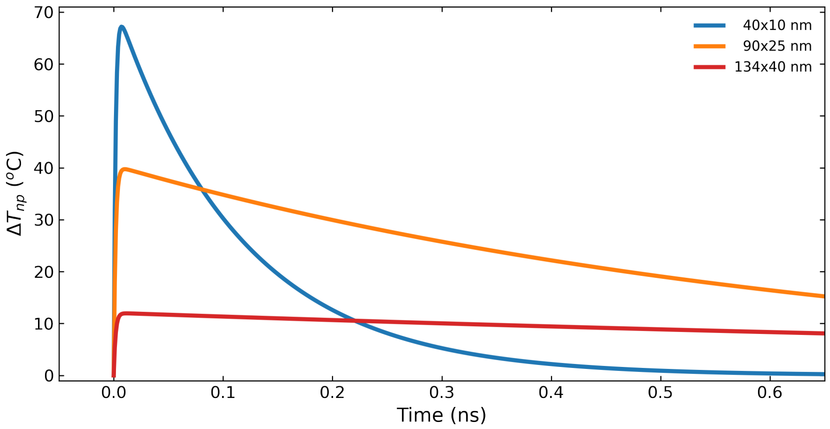

Figure S3. Temperature dynamics of a single gold nanorod of sizes 40x10nm (blue), 90x25nm (orange) and 134x40nm (red). Laser intensity was 1000mW/mm^2 at 808nm.

The average temperature of a single NP under fs laser pulses is obtained by taking the average integral over the laser repetition rate period:

$$\langle \Delta T_{np}^{fs} \rangle = \frac{\langle I \rangle \tau_d}{\rho_{Au} c_{pAu}} \frac{\sigma_{abs}}{V_{np}} \left[1 - \frac{e^{-1/f\tau_{ep}}}{1 - \tau_{ep}/\tau_d} \right]. \tag{S6}$$

4. COLLECTIVE HEATING OF NANOPARTICLES UNDER DIFFERENT LASER IRRADIATION REGIMES

A. CW Irradiation

The global temperature variation of a colloidal sample in the steady-state (ΔT_{global}) is given by:

$$\Delta T_{global}^{CW} = \sum_{n=1}^{\#_{np}} \frac{\sigma_{abs} I}{4\pi\kappa_m \beta |\vec{r} - \vec{r}_n|}, \tag{S7}$$

If we consider that the macroscopic volume is thermalized, it is possible to exchange the contribution of each individual NP by a homogeneous heat source for irradiation region ($\#_{np} = C_{np}V$). Therefore:

$$\Delta T_{global}^{CW} = \frac{C_{np}\sigma_{abs} I}{4\pi\kappa_m \beta} \int_V \frac{dV'}{r'}. \tag{S8}$$

However, $\Delta T_{np}^{CW} = \sigma_{abs} I / 4\pi\kappa_m \beta R_{eq}$, which leads to:

$$\Delta T_{global}^{CW} = C_{np} \Delta T_{np}^{CW} R_{eq} \int_V \frac{dV'}{r'}. \tag{S9}$$

B. Femtosecond Irradiation

Following a similar procedure, we start from the superposition of each individual NP. However, since we're dealing with steady-state cumulative heating, we must consider the average temperature of a single NP over the repetition rate period:

$$\Delta T^{fs}_{global} = \sum_{n=1}^{\#_{np}} \frac{\langle I \rangle \tau_d}{\rho_{Au} c_{pAu}} \frac{\sigma_{abs}}{V_{np}} \left[1 - \frac{e^{-1/f\tau_{ep}}}{1 - \tau_{ep}/\tau_d} \right]. \tag{S10}$$

Using the same consideration described in the previous section and using $V_{np} = 4\pi R^3_{eq}/3$, Equation S10 can be written as:

$$\Delta T^{fs}_{global} = \frac{3C_{np}\ \sigma_{abs} \langle I \rangle \tau_d}{4\pi \rho_{Au} c_{pAu}} \left[1 - \frac{e^{-1/f\tau_{ep}}}{1 - \tau_{ep}/\tau_d} \right] \int_V \frac{dV'}{r'^3}\ , \tag{S11}$$

and similarly:

$$\Delta T^{fs}_{global} = C_{np} \langle \Delta T^{fs}_{np} \rangle V_{np} \left(\frac{3}{4\pi} \int_V \frac{dV'}{r'^3} \right)\ , \tag{S12}$$

5. RAW THERMAL EXPERIMENTAL RESULTS

Sample heating of 400μL of specimen in 2mm cuvette was performed under 808nm laser illumination with 1.5W of optical power reaching the samples. The samples were irradiated for 20min and left cooling down 20min. This process was repeated three times for each sample. Figures S4, S5 and S6 illustrates the results obtained in the first, second and third measurement respectively.

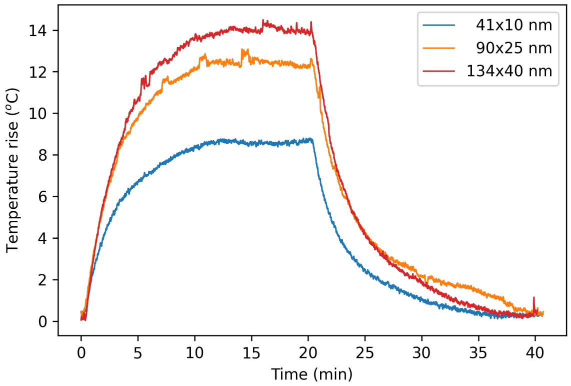

Figure S4. First sample measurement. Blue: 40x10nm, Orange: 90x25nm and Red: 134x40nm.

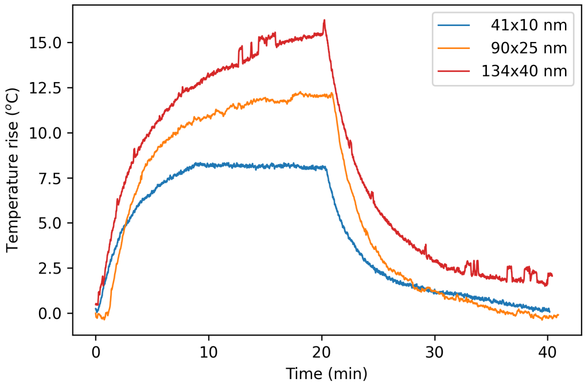

Figure S5. Second sample measurement. Blue: 40x10nm, Orange: 90x25nm and Red: 134x40nm.

REFERENCES

1. K. L. Kelly, E. Coronado, L. L. Zhao, and G. C. Schatz, "The optical properties of metal nanoparticles: the influence of size, shape, and dielectric environment," The J. Phys. Chem. B **107**, 668–677 (2002).

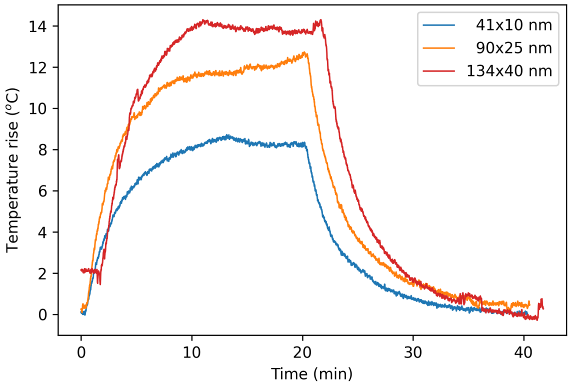

Figure S6. Third sample measurement. Blue: 40x10nm, Orange: 90x25nm and Red: 134x40nm.

2. G. Baffou, *Thermodynamics of Metal Nanoparticles* (Cambridge University Press, 2017), p. 36–80.
